# Quality of life in pediatric intestinal failure: A scoping review

**DOI:** 10.1016/j.intf.2025.100071

**Published:** 2025-07-10

**Authors:** Fatima Wasif, Dylan Johnson, Anna Gold

**Affiliations:** a Department of Psychology, University of Waterloo, 200 University Ave W, Waterloo, ON N2L 3G1, Canada; b Department of Applied Psychology and Human Development, University of Toronto, 252 Bloor St West, Toronto, ON M5S 1A1, Canada; c Department of Psychology and Transplant and Regenerative Medicine Centre, The Hospital for Sick Children, 555 University Ave, Toronto, ON M5G 1X8, Canada

**Keywords:** Quality of life, Intestinal failure, Pediatrics, Measurement, Scoping review

## Abstract

**Background:**

Interest in quality of life (QoL) of children with intestinal failure (IF) has increased in recent years, driven by improved survival rates. Conceptualization and measurement of QoL remains understudied. As such, we conducted a scoping review to systemically catalogue measures of QoL, collate existing findings about QoL among IF patients, and examine risk and protective factors.

**Methods:**

A systemic search was conducted across four databases (MEDLINE, EMBASE, PsychINFO, CINAHL) from inception to May 6, 2024. Included articles had to explicitly operationalize and measure QoL among pediatric IF patients (< 18 years of age).

**Results:**

Thirty-three articles met inclusion criteria from an initial screen of 2124. Across studies, QoL was most frequently measured through standardized questionnaires, with the PedsQL most frequently employed. On self-report, patients reported worse health-related QoL, as well as lower physical, social, and school functioning, compared to controls. Adolescents with IF reported worse QoL than children. Youth and caregivers diverged in their reporting on QoL, with caregivers reporting worse overall QoL outcomes than children. Medical (bowel functioning), social (participation in activities with peers), and familial (parental stress and anxiety) risk and protective factors also emerged.

**Conclusions:**

IF has been found to have a negative impact on QoL for children and caregivers. It will be important to establish a standardized IF-specific QoL questionnaire to capture IF-relevant medical and treatment parameters, as well as include an evaluation of physical, emotional, social, psychological, and school functioning. Our findings help lay groundwork for establishing clinical guidelines for patient assessment and monitoring.

## Introduction

Intestinal failure (IF) is the reduction of functional gut mass under the level required to maintain normal growth and fulfill daily energy and fluid requirements through enteral nutrition [1]. The consensus definition of IF specifies length of dependence on supplemental parenteral nutrition (PN) support as being a minimum of 60 days within a 74 consecutive day interval [2]. The most common causes of pediatric IF are short bowel syndrome (SBS), motility disorders, and mucosal enteropathies. Due to the implementation of multi-disciplinary intestinal rehabilitation teams and liver preservation strategies [3], survival rates have increased significantly in the past decade, from 60 % to up to 95 % [4].

### Rationale

Despite higher rates of survival, pediatric IF is associated with numerous clinical complexities. These include early and frequent hospitalizations, multiple surgical interventions, reliance on long-term PN, and the risk of infection and related comorbidities, such as intestinal failure associated liver disease or organ transplantation [5]. Evidence has also suggested greater challenges with physical, motor, cognitive, and psychological functioning in pediatric IF patients, relative to healthy peers and other pediatric chronic disease groups [6]. With improved survival, there are increased risks for longer-term morbidity among children with IF, which likely impacts their Quality of Life (QoL). The most widely cited definition of QoL is “an individual’s perception of their position in life in the context of the culture and value systems in which they live and in relation to their goals, expectations, standards and concerns” [7]. This World Health Organization definition encompasses specific domains of functioning, disability, and health [8].

Outcome metrics need to consider the broader concepts of health that reflect overall QoL and well-being, as well as incorporate self-reported evaluations of QoL to adequately capture patient experience and optimize functioning. Research has examined QoL among other chronically ill populations, including pediatric cancer [9], [10], stroke [11] and transplant patients [12]. Generally, these groups are at a greater risk for challenges across multiple domains of functioning, including education, employment, and peer relationships. Notably, outcomes are influenced by several variables, including medical (e.g., pain, fatigue, treatment burden), psychological (e.g., depression, resilience), and socio-demographic (e.g., gender, education, financial status) factors. The complex interactions between risk and protective factors in predicting patient outcomes show the need for collaboration among different professionals to better understand the QoL of people with chronic illness, including children with IF.

A better understanding of long-term QoL in pediatric IF can assist in treatment planning and clinical interventions, as well as inform decision-making regarding which multidisciplinary team members should be integrated into clinical care. QoL outcomes remain understudied in pediatric IF, and to our knowledge, have not been systemically evaluated. Further, interdisciplinary collaboration necessitates uniformity in QoL measures across teams. With little systematic evidence collating approaches to QoL measurement in pediatric IF patients, there is a gap that must be addressed in order to optimize long-term care for these patients.

### Objectives

The current study is a scoping review investigating QoL in pediatric IF. We had four primary objectives. First, to systemically catalogue primary studies of QoL in children with IF. Second, to systemically collate measures of QoL used in pediatric IF research and respective domains of QoL captured. Third, to synthesize findings about the links between QoL and pediatric IF. Fourth, to account for potential risk and protective factors which may moderate the association between pediatric IF and QoL.

## Methods

### Protocol and registration

The study protocol was pre-registered on the Open Science Framework (OSF) database (https://osf.io/cm5xn). Reporting for this scoping review was guided by the Preferred Reporting Items for Systematic reviews and Meta-Analyses extension for Scoping Reviews (PRISMA-ScR) Checklist [13].

### Eligibility criteria

Eligibility criteria were established a-priori and included the following: Pediatric patient populations (age 0–17.99 years) who were diagnosed with IF and assessed for QoL (directly or by proxy via a caregiver). Exclusion criteria included non-English language articles, non-peer reviewed documents (e.g., book chapters, dissertations, and conference proceedings), non-primary studies (e.g., reviews), adult populations (>18 years of age), intestinal transplant only populations, and if QoL measures were not incorporated. QoL measures were deemed acceptable for inclusion via formal (e.g., PedsQL) and informal methods (e.g., qualitative assessment), if the authors explicitly stated QoL was the construct they were operationalizing.

### Information sources

A systematic search was conducted in MEDLINE (Ovid system), EMBASE (Ovid system), PsycINFO (Ovid system), and CINAHL databases to carry out a scoping review interested in direct applications of QoL measures in pediatric IF populations. In order to focus on high-quality, peer-reviewed research, grey literature sources (e.g., pre-prints, dissertations, and government reports) were not considered.

### Search

In collaboration with a research librarian, a search strategy was designed for implementation in the previously mentioned databases (Supplemental Table 1). The broader search constructs that were represented included the exposure (i.e., intestinal failure), outcome (i.e., quality of life), and the population (i.e., pediatrics). Keywords and Medical Subject Headings (MeSH) terms were used when conducting the search strategy. The search strategy was initially developed on EMBASE and then keywords/MeSH terms were adjusted accordingly for the other databases. The final search strategy was conducted on May 6, 2024.

### Selection of sources of evidence

A blinded, dual screening process was conducted at the abstract, full-text, and extraction phases of the study. Before initiating screening, a review of the selection criteria was conducted, and a sample of abstracts (*n* = 30) was piloted until a sufficient level of agreement (Cohen’s kappa (κ) > .8) was observed between all three screeners. At each stage of the process, conflicts between screeners were addressed via consensus meetings. The study article selection phase of the project was managed with Covidence software. A backwards citation search was conducted on all included articles, whereby the authors reviewed the reference lists to identify relevant articles.

### Data charting process

A blinded, dual extraction process was conducted, where each author was randomly provided a list of articles to extract from using a provided extraction sheet (Microsoft Excel). The extraction sheet included details pertaining to the study population, presenting medical exposures, and details about QoL measures and relevant research findings. Following the independent extraction of each manuscript, one author compared spreadsheets and consolidated extracted information into a final data sheet. Conflicts were discussed as a team and agreed upon via consensus.

### Data items

Extracted items included participant baseline characteristics (age, sex, and ethnicity), sample size, country/region, study design (e.g., cross-sectional or longitudinal), time-at-risk (i.e., time elapsed between exposure and outcome), measures of QoL used, risk and protective factors for QoL, and observed relationships or descriptions pertaining to QoL in pediatric IF populations.

### Synthesis of results

In this scoping review, a qualitative synthesis of included studies was conducted. This included multiple objectives. First, the objective of organizing and cataloging all research conducted on QoL in pediatric IF, extracting relevant demographic, methodological, and analytic findings into tables and text. Next, all measures of QoL identified in our investigation were documented alongside descriptions of medical and psychosocial factors captured in each measure. Finally, a narrative synthesis was conducted to describe the main themes and trends identified through the investigation, as well as recommendations for how stakeholders (e.g., clinicians and researchers) can improve and standardize approaches towards documenting QoL in pediatric IF.

## Results

### Selection of sources of evidence

**Fig. 1:**
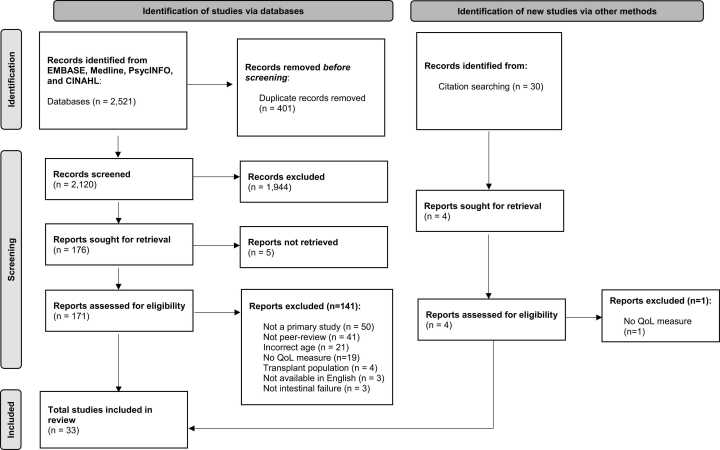
PRISMA flow diagram.

Source: Page MJ, et al. BMJ 2021;372:n71. doi: 10.1136/bmj.n71. This work is licensed under CC BY 4.0. To view a copy of this license, visit : https://creativecommons.org/licenses/by/4.0/

A search of Medline, PsycINFO, EMBASE, and CINAHL was conducted returning a total of 2,521 articles (Fig. 1). Removing duplicates (*n* = 401), 2,120 articles were screened as abstracts. Abstract screening identified 176 articles which were screened as full-texts, where a total of 30 articles were included in the final review. Citation review of these 30 articles yielded four additional studies which were screened for inclusion, with three included in the final review. The final scoping review comprised a sample of 33 articles [14], [15], [16], [17], [18], [19], [20], [21], [22], [23], [24], [25], [26], [27], [28], [29], [30], [31], [32], [33], [34], [35], [36], [37], [38], [39], [40], [41], [42], [43], [44], [45], [46]. Inter-rater reliability was excellent across reviewers (κ = 0.80–1.00).

### Characteristics of sources of evidence

Sociodemographic, medical, and methodological results are presented in Table 1. Included studies examined QoL within pediatric IF samples primarily across Europe (*n* = 22 studies; *n* = 9 from the Netherlands) and North America (*n* = 9 studies; *n* = 8 from the United States of America). Twenty-three studies were published in the last 10 years. Combined across studies, the overall pediatric IF sample size was *n* = 1,732. The most represented IF aetiologies were motility disorders (primarily Hirschsprung’s Disease (HD; *n* = 958)) and SBS (*n* = 656; most commonly Necrotizing Enterocolitis or NEC at *n* = 173). Most studies compared patient outcomes to controls (*n* = 28). Control samples were comprised of healthy controls (*n* = 12 articles) or children or caregivers of children experiencing chronic illness (*n* = 5 articles), such as inflammatory bowel disease, diabetes mellitus, or juvenile rheumatoid arthritis. Comparisons were also made to sample norms or population level normative data (*n* = 15 articles). Ages ranged from 0.5 to 18 years old and comprised a majority of male patients (*n* = 27 articles).

**Table 1:** Quality of life in pediatric intestinal failure patients.

**Reference**	**Full sample characteristics**	**Exposed sample characteristics**	**Control sample characteristics**	**Patient diagnosis (N)**	**Quality of life measure**	**Relevant findings**
Bai et al., 2002 (China) [14]	N = 89 (17.7 % female)	N = 45 (17.7 % female; M_age_=10.9 [8−16])	N = 44 (age and sex similar to IF children)	HD^a^ (45)	QoL scoring criteria for children with fecal incontinence	Overall, IF children reported lower QoL than controls (*p* < .05; 7.7; *SD*=2.9 compared to 11.6; *SD*=0.7), despite most children (86.7 %) endorsing good to fair QoL. Persistent fecal soiling or incontinence after surgery was linked to poorer social and emotional functioning outcomes (e.g., school absence, peer victimization) among these school-age children.
Belza et al., 2023 (Canada) [15]	N = 62 (50 % female)	N = 34 (47.1 % female; M_age_=5.1, *SD*=4.4)	N = 28 (53.6 % female; M_age_=6.4, *SD*=5.0)	Abdominal wall defect (9) Atresia (3) HD (4) NEC^b^ (5) Volvulus (2) Other (11)	PedsQL Family Impact Module (FIM)	Children were noted to have significantly lower physical, emotional, social, cognitive and communicative functioning (*ps* <.01). Caregivers reported significantly more worry and stress than controls, with a strong significant correlation between stress and worse health-related QoL. Potential sources of parental stress remained unexplored.
Brooks et al., 2020 (United States of America) [16]	N = 67 at baseline (26.9 % female)	N = 45 (13.3 % female; M_age_=6.5 [3−10])	54.5 % female	Rectosigmoid HD (67)	Rintala BFS	Post-surgery, median bowel-functioning related QoL was 12 [range:8−19], with notable child reporting of greater social problems (median=3; range [1 - 3]). Decreasing ganglion size and increasing neurotransmitter expression were linked to lower bowel-functioning QoL scores.
Bystrom et al., 2021 (Sweden) [17]	N = 60	N = 30 (26.9 % female; Median_age_=7 [4−11])	N = 30 (age-and-sex-matched)	HD – Long segment (1) HD – Short segment (29)	KIDSCREEN−52	QoL did not differ significantly between children with HD and controls on any domains except financial resources, with HD child households reporting less income than control families (*p* = .008), despite income compensation provided to their families. HD children did endorse more social problems than controls (30 % compared to 0 %), and notably that they experienced foul odours.
Davidson et al., 2022 (Finland) [18]	N = 84 (21.4 % female)	N = 84 Duhamel: 22 % female; M_age_= 11 [5−18] ERPT: 10.5 % female; M_age_= 10 [4−18])	Unpublished, normative data comparison	HD (84)	Peds QL Rintala BFS	Overall, bowel-functioning related QoL was lower in both Duhamel and ERPT cohorts compared to controls (*ps*<.001). Poor bowel functioning, including poor fecal control, soiling frequency, and accidents, was linked to consistently lower QoL. Children who underwent the Duhamel procedure fared worse than the ERPT cohort and controls in terms of experienced social problems (*p* < .001).
Edge et al., 2012 (United Kingdom) [19]	N = 19 No further descriptives.	N = 19		Gast/Volvulus (1) Gast/atresia (3) Gastroschisis (2) HD (1) Jejunal Atresia (2) NEC (7) Vanishing Gast (1) Volvulus (2)	SBS-relevant QoL survey adapted from prior research	Post-surgery, caregivers reported a consistent and significant improvement in their child’s development, physical, and bowel functioning, as well as their life enjoyment. Caregivers noted QoL improvement as a vital expected outcome of surgery, although QoL was not directly measured in a standardized manner in the study. Caregivers also reported increased quality of time with children, as well as greater optimism for the future post-intervention, noting that their child’s needs were easier to manage after surgery.
Emedo et al., 2010 (United Kingdom) [20]	N = 7 (86.7 % female; [7−17])	N = 7 (86.7 % female; [7−17])		Extreme or Complex SBS (3) Myopathic IPO (1) Severe Congenital Enteropathies (3)	Focus Interviews	Themes centered around exploring QoL in patients on home parenteral nutrition. Most children expressed that they were able to tolerate life on parenteral nutrition. Anaccurate understanding of their illness was spoken of as conducive to their emotional functioning and health-related QoL. Children noted restrictions to their physical activity, especially outside the home, as challenging. Further, children indicated wanting to fit in with peers, with friends typically noted to be accepting and supportive of their peer with intestinal failure. QoL was considered diminished among these children due to the interference of parenteral nutrition in limiting participants ability to achieve personally relevant goals.
Hartman et al., 2007 (Netherlands) [21]	N = 445 total questionnaires	N = 316 (first set); N = 250 (second set) (ARM children: 32 % female; M_age_=9.5, *SD*=1.1, ARM adolescents: 26 % female; M_age_=13.9, *SD*=1.5; HD children: 25 % female; M_age_=9.2, *SD*=1.1, adolescent HD: 11 % female; M_a__ge_=14, *SD*=1.4).		Children: ARM^c^ (76), HD (65) Adolescents: ARM (53), HD (56)	TNO-AZL TACQoL child and adolescent forms HAQL	Overall, QoL did not differ significantly between children with ARM and HD. Children reported improvements in their disease-specific functioning over-time (d=.22, *p* < .01), and their psychosocial functioning (d=.15, *p* = .01). Physical and mental QoL predicted one another over the study time-points. Individual variation within the patient groups was observed, in that children with HD and ARM reported lower perceived competency than healthy controls, compared to adolescents, who reported their global self-worth as greater than their healthy counterparts. The strongest predictor for QoL was determined to be self-competence, especially within the domain of school attitude.
Hartman et al., 2008 (Netherlands) [22]	N = 2125	N = 164 ARM, 152 HD (HD: 22 % female children, M_age_=9.2 [8−12], 18 % female adolescents, M_age_=14 [12−17])	N = 1809 (Children: [8−11] Adolescents: [12−15])	ARM (164) HD (152)	TNO-AZL TACQOL HAQL	Children with ARM and HD reported significantly lower perceived self-competency than healthy peers (*p* < .001), although QoL did not differ between the two patient groups. Adolescents with IF reported higher self-esteem in relation to their physical appearance (*p* < .01). Adolescents with HD noted better global self-worth (p = .01), while adolescents with ARM reported better social acceptance (*p* < .01) and athletic competency (*p* < .001).
Hartman et al., 2015 (Netherlands) [23]	N = 98 caregivers N = 44 children with ARM N = 54 children with HD (35.2 % female total)	N = 98 (25 % female; M_age_=11.2)	N = 98 healthy siblings (46 % female; M_age_=12.5)	ARM (44) HD (54)	Health-related QoL TNO-AZL TACQOL Both self-and-parent report versions	Children with ARM or HD reported worse physical functioning (more pain and symptoms; *p* = .002) and less positive emotions (*p* = .027) than their caregivers. The older the child, the better their reported social functioning (*p* = .032), and the lower their experienced negative affect (*p* = .003). Parent reflections on the QoL of their healthy children did not differ among domains of QoL. Caregivers of patients with ARM and HD, therefore, tend to overestimate their child’s health-related QoL. However, parent reporting was closely aligned with child reports within the domains of cognitive and social functioning.
Hijkoop et al., 2019 (Netherlands) [24]	N = 137	N = 31 (45 % female; Median_age_=9, IQR 6–13, [4−16])	N = 46 for PedsPCF, 60 for PedsQL and DUX−25 (age-sex-maternal education matched)	Simple Gastroschisis (27) Complex Gastroschisis (3)	PedsQL DUX−25	Children reported total QoL scores that did not differ significantly from those of matched controls (*p* = .82, median_patient_=86 compared to median_control_ 84), except in the domain of school functioning. School functioning was significantly lower in children than controls (*p* = .04; median_patients_=78 compared to median_controls_ =80), although this difference was not considered clinically relevant. Older children (8−17) with gastroschisis reported considerably worse physical and home functioning than controls (p = .03 and p = .04, respectively), which were thought to be related to factors like negative self-image due to impaired physical growth.
Hilberath et al., 2024 (Germany) [25]	N = 93 caregivers (72 % female; [16−70])	N = 44 children with IF (40.9 % female; M_age_=7.4, Median_age_=6.0)	Caregivers of children with IBD (N = 18) or healthy controls (N = 31) IBD patients: (38.9 % female; M_age_=10.9, Median_age_=12.0) Healthy controls: (16.1 % female; M_age_=6.8, Median_age_=6.5)	SBS (29) Mucosal Enteropathy (4) PIPO^e^(11)	Unstandardized QoL questionnaire targeted at evaluating the impact of the COVID−19 pandemic on QoL	Caregivers reported an increased deterioration in their child’s QoL during the COVID−19 pandemic, across groups. Over eighty percent (81.8 %) of children with IF noted worse QoL during the pandemic. The disruption in provision of medically necessary care, such as medical aids and parenteral nutrition negatively impacted child health.
McCaig et al., 2021 (United States of America) [26]	N = 9769	N = 53 (50.9 % female; M_age_=6.2, *SD*=3.9)	N = 9430 healthy controls, 286 chronic gastrointestinal patients	Gastroschisis (13) HD (5) Intestinal Atresia (18) NEC (15) Volvulus (13) Other (8)	PedsQL Generic Core PedsQL GI Symptoms Module	Children with IF and their caregivers reported lower disease-specific health-related QoL compared to healthy controls. In contrast, generic QoL is not significantly lower in IF children compared to controls (*p* = .07) when reported by caregivers but is considerably lower than healthy controls when explored via self-report (*p* < .001). Across children and caregivers, school and emotional functioning is lower than that of healthy controls, with IF patients often (81 %) attending school with specialized education plans.
Meinds et al., 2019 (Netherlands) [27]	N = 320 (20.8 % female; Median_age_=18 for combined adult and child sample)	N = 173 children ([8−17])	N = 147 (age-and-sex-matched controls)	HD (173)	CHQ – Child Form	Children with HD had significantly lower QoL than age-matched norms, with lower self-esteem (*p* < .008), greater behavioural difficulties (*p* < .001), and poorer general health (*p* < .001). Bowel problems, including constipation and fecal incontinence, was linked to negative QoL outcomes among IF children.
Mutanen et al., 2015 (Finland) [28]	N = 99	N = 36 (36 % female; Median_age_=9; in analysis [7−18])	N = 63 (40 % female)	Midgut Volvulus (7) NEC (11) Small Bowel Atresia (5) Extensive HD (10) CIPO^d^ (3)	PedsQL Generic Core Scales	Self-reported physical functioning was significantly lower in IF children compared to controls, with physical and school functioning correlated to the severity of intestinal failure children reported (*r* = .615, *p* = .02 and *r* = .706, *p* = .03, respectively. Caregiver report suggested that caregivers tend to estimate lower health-related QoL than children, with the greatest difference observed in emotional functioning between the two groups. The provision of parental support to ease stress was suggested.
Nagelkerke et al., 2022 (Netherlands) [29]	N = 35 (40 % female; Median_age_=7.9, IQR 5.5–13)	N = 35 (40 % female; Median_age_=7.9, IQR 5.5–13)	Compared to normative Dutch population	Congenital Enteropathy (4) Intestinal Neuromuscular Motility Disorder (12) SBS (15) Other (4)	PedsQL Generic and Fatigue on the KLIK [Dutch Acronym for Quality of Life in Clinical Practice]	QoL was reported as worse than the reference population among young children, aged 5–7 (d=.73), along with uniquely greater sleep fatigue. Similar patterns were observed in school age-children (8–12; d=.71). In older children (13−18), only physical functioning was significantly lower compared to controls.
Neam et al., 2020 (United States of America) [30]	N = 378	N = 91 (38 % female; Median_age_=3, [0.5–17])	N = 246 healthy controls, 41 chronically ill children	Gastroschisis (29) HD (5) Intestinal Atresia (11) Midgut Volvulus (7) NEC (26) Other (13)	PedsQL PedsQL Generic Core Scales	Children experiencing IF were reported to have lower total health-related QoL scores compared to healthy controls, with school-aged children also observed to have lower QoL when compared to chronically ill controls. School-related factors were thought to be considerably impactful in reduced QoL for school-age children with intestinal failure, given that their school functioning is significantly lower compared to even chronically ill controls (difference 25.6, *p* < .001). QoL declined as children grew older, with decreases in QoL thought to be linked to lower school functioning.
Neam et al., 2022 (United States of America) [31]	N = 1022 (combined pediatric and caregiver sample)	N = 93 (39.3 % female; Median_age_=3, [0.5–17])	N = 929; 546 healthy children and 383 chronically ill children	Gastroschisis (38) NEC (31) Intestinal Atresia (17) Congenital Short-Bowel (2) HD (7) IPO^e^ (4) CIPO (3) Meconium Peritonitis (3) Midgut Necrosis (1) Midgut Volvulus (9) MID^f^ (2)	PedsQL 2.0 FIM PedsQL Generic Core Scales 4.0	Family-related QoL was reported to be significantly lower in families with children experiencing IF (mean difference= −4.9, *p* = .01), but like families with children who are chronically ill. Compared to healthy and chronically ill controls, IF families reported significantly lower communication effectiveness and higher worry (*ps* <.001), but higher family functioning (*p* = .004). Parenteral dependence was noted to interfere in daily activities and communication, like engaging in family activities or chores.
Neuvonen et al., 2017 (Finland) [32]	N = 156 (26 % female, Median_age_=15, [4−32] combined pediatric and adult sample).	N = 39	N = 117 (age-and-gender matched)	HD (39)	PedsQL	IF children less than 8-years-old had more frequent loss of educational opportunities than controls (*p* < .05), such as through school absence. For children aged 8–18 years, QoL was not significantly lower than controls, with greater reported emotional and social functioning than the control sample (*p* < .05). This may be due to the development of robust coping strategies over the course of illness. Child-reported QoL differed from patients in that caregivers reported that their children were able to keep up as well as their peers at school and were less depressed than what children self-reported.
Olieman et al., 2012 (Netherlands) [33]	N = 306	N = 31 (61 % female; M_age_11.3, *SD*=4.1)	N = 275 community healthy controls (58 % female; Mage=11.2, *SD*=3.2)	Small Bowel Atresia (12) NEC (7) Meconium Peritonitis with CF (4) Gastroschisis (3) Volvulus and/or Malrotation (2) Meconium Peritonitis with no CF (4) Long-Segment HD (1) Ischemic Small Bowel (1)	PedsQL	Children with SBS reported significantly lower health-related QoL compared to healthy-controls (*p* = .01). SBS children also reported lower psychosocial health scores (*p* = .01) than healthy-controls, as well as lower physical (*p* = .02) and school functioning (*p* = .03). At the same time, emotional (*p* = .16) and social functioning (*p* = .10) did not differ significantly between SBS children and controls. Older control participants (11 and older) had significantly higher scores (*p≤*.02) than younger controls in terms of health-related QoL. Adolescents with SBS fared worse than SBS children in terms of their QoL, which was attributed to the unique challenges of adolescence. The authors speculated that this age-group may have greater awareness of their disease, and the bodily changes that accompany it, making them prone to feelings of low self-esteem. Caregiver-proxy scores for patients were significantly lower than controls (p < .01), with socioeconomic status a significant effect modifier on all QoL scales (except emotional).
Pederiva et al., 2019 (United Kingdom) [34]	N = 53	N = 30 Under five-years-old: N = 17 (47.1 % female; M_age_=3, Median_age_=3.1, [0.5–5]). Over five-years-old: N = 13 (53.4 % female; M_age_=10.9, Median_age_=8.9, [5−18]).	N = 5962 healthy-controls, 12 Children’s Convalescent Hospital, 11 from REACH sample	Under five-years-old: Volvulus in Malrotation (10) Gastroschisis (3) NEC (2) Volvulus in Mesenteric Lymphangioma (1) Intestinal Atresia (1) Over five-years-old: NEC (3) Gastroschisis (5) Intestinal Atresia (3) Volvulus in Malrotation (1) Cloacal Exstrophy (1)	Sent to all: PedsQL FIM PedsQL Healthcare Satisfaction Generic Module Sent to patients older than five: PedsQL General Well-Being Scale PedsQL 4.0 Generic Core	Comparing SBS children above five to healthy controls, SBS children scored worse on all metrics of health-related QoL (*p* < .001), including physical (*p* = .002), psychosocial health (*p* = .002), social (*p* = .001), and school functioning (*p* = .01). Emotional functioning did not differ significantly between the samples (*p* = .37). SBS families scored worse than control families from the CCH sample in terms of their family functioning, which included activities of daily living and family relationships (*p* = .003). Families of SBS children over the age of five reported a significantly higher family functioning score than those under five (*p* = .03). Children with SBS and those in long-term care were rated poorly by their families in terms of functional independence. Further, most children reported medium-high well-being, and medium health.
Proli et al., 2021 (France) [35]	N = 45 sampled, 31 included in final analysis. (32.2 % female)	N = 8 (on home parenteral nutrition) (13 % female; Median_age_=12; IQR: 10–14)	N = 13 intestinal transplant (IT), 10 liver transplant (LT) (Median_age_=15) IT: 30 % female; IQR (14−18) LT: 50 % female; IQR (14−17)	SBS (13) Motility Disorder (4) Congenital Enteropathy (4)	CHQ-CF87 French Version CHQ-PF50 French Version	IF children receiving home parenteral nutrition reported significantly worse global health (*p* = .04) compared to intestinal transplant patients. However, caregivers of intestinal transplant children noted lower health-related QoL in multiple domains, including emotional and behavioural difficulties, compared to children on home parenteral nutrition.
Sanchez et al., 2013 (United States of America) [36]	N = 1127	N = 23 (30.4 % female; M_age_=28.5 months, *SD*=16.9 months).	N = 1104	NEC (10) Gastroschisis (8) Congenital SB (1) HD (1) Intestinal Atresia (1) Meconium Peritonitis (1) Midgut Volvulus (1)	PedsQL 4.0 Generic Core Scales PedsQL Infant Scales PedsQL FIM “What other things affect your child’s and/or your family’s quality of life not asked in this questionnaire?’ ‘How do these other things affect your child’s and/or your family’s quality of life?’	Children with iIF, across ages, showed lower overall health-related QoL than healthy children, according to their caregivers, with physical and psychosocial functioning notably lower in IF children (*ps* <.001). In addressing the PedsQL’s ability to capture their child’s QoL, 65 % of caregivers noted that the survey did not address vital aspects of intestinal failure, such as decreased mobility, and the burden of central lines. These concerns were subsumed under the theme of the complexity of intestinal failure interfering with caregivers’ ability to provide nurturing care to their children.
Schwankovsky et al., 2002 (United States of America) [37]	Unreported for control population	N = 45 (53.3 %female; M_age_=9, [1−21])	Normative data for children with juvenile rheumatoid arthritis and healthy-controls	CIPO (45)	CHQ	Children with CIPO reported lower levels of self-care, mobility, ease of school attendance, and social activity participation than healthy controls (*p* < .001). CIPO children had similar self-care and mobility as chronically ill controls (77 average), but lower school attendance and social activity participation (*p* < .001). Older children with CIPO were noted to have lower self-esteem (*p* < .001) and greater levels of depression and anxiety (*p* < .05) than younger CIPO children. Significant associations between the ability to participate in school / social opportunities and child mobility (*p* < .001), pain levels (*p* < .001), and mental health also emerged (*p* < .05). Caregivers reported greater emotional difficulties and burden of care compared to control groups.
Silva et al., 2022 (Portugal) [38]	N = 20	N = 20 (50 % female; M_age_=7.5, *SD*= 5.0, [1.3–19.2])	Compared to healthy and chronically ill (spina bifida and diabetes mellites) children	SBS^g^ (12) Ultra-SBS (8)	PedsQL Generic Core Scales	Average PedsQL scores for children with SBS were slightly lower compared to normative scores in the Portuguese population. Big reductions were observed in the school functioning domain for children with SBS, even compared to children with chronic diseases like diabetes. Challenges with school functioning were also reported by IF caregivers, highlighting the negative role of school absence to QoL within this demographic.
Spagnuolo et al., 2013 (Italy) [39]	N = 28	N = 16 (25 % female; M_age_=7.2, *SD*=4.8)	N = 12 healthy controls (M_age_=9.3, *SD*=4.9)	SBS (13) Shwachman Syndrome (1) HD (1) Primary Fatty Acid Malabsorption (1)	ICF-CY STAI-S PGWBI TMA	Caregiver and child QoL were noted to be highly negatively impacted by lack of availability of support from relatives (*p* = .002) as well as care providers. Children did not differ significantly in their self-esteem from controls, although healthy controls scored higher in terms of their self-esteem within the school setting (M_difference_=−14) and in the home (M_difference_=−13). Although caregivers noted well-being in the high-good range, 31 % of parents endorsed medium or high levels of anxiety.
Tran et al., 2019 (United Kingdom) [40]	N = 17 (21 % female)	N = 14 (Median_age_=4, [1−17])		Dysmotility (6) MID (1) SBS (10) *3 participants were not retained for analysis	Baxter HPN-QoL (reduced from 48 to 46 questions)	The QoL of children on home parenteral nutrition was reported to be good, with an overall score of 8 out of 10, with 10 representing best QoL. Caregivers reported their children were able to engage in social activities and work through daily living activities, given they were able to rest. Still, learning at school, maintaining a social life, and freedom of movement were identified as significant areas of difficulty for children on home parenteral nutrition.
van Oers et al., 2019 (Netherlands) [41]	N = 62 caregivers, 37 children	N = 37 mothers, 25 fathers, 37 children Children: 45.9 % female; patients: M_age_= 5.1, SD= 4.6, Median_age_= 3.6, [3–17.4])	Dutch caregivers of healthy children	Chronic IPO (12) SBS (8) Intestinal Obstruction due to Congenital Malformations (7) MID (4) Chronic Intractable Diarrhea (4) HD (1) Severe Failure to Thrive (1)	TNO-AZL TAAQOL	Caregivers of children on home parenteral nutrition did not differ significantly from caregivers of healthy children on any health-related QoL dimensions except for significantly lower depressive emotions for mothers (median score 75 for IF mothers compared to 83 for reference mothers, *p* = .01) and activities of daily living for fathers (median score 88 for IF fathers compared to 100 for reference fathers, *p* = .04). Caregivers reported that their children had difficulties with being independent, as well as struggled in terms of social functioning (greater loneliness and less leisure time).
Vlug et al., 2023 (Netherlands) [42]	N = 41 (54 % female; Median_age_=8.9, IQR: 5.5–11.8)	N = 41 (54 % female; Median_age_=8.9, IQR: 5.5–11.8)	Compared to normative data; no other descriptives provided	NEC (14) Other (8) Intestinal Atresia (5) IPO Syndrome (5) Midgut Volvulus (5) Gastroschisis (4)	PedsQL Gastrointestinal Symptoms Module	Lower gastrointestinal-related QoL was related to more reported emotional challenges in IF children, which was thought to reflect the connection between the gut and the brain in promoting psychological health.
Weih et al., 2010 (Germany) [43]	N = 131 sampled	N = 77 completed interviews for outcomes (45.8 % female; <1 year at time of surgery).		Intestinal Atresia (34) NEC (21) Meconium Ileus (18) HD (18) Volvulus (18) Other (22)	No singular standardized QoL measure. Restrictions in daily life Effect on family life (OP) Effect on family life (NOW) State of health Development	Post-intestinal anastomosis, children were reported to have no restrictions in daily life or disruptions, with only 7.3 % of caregivers reporting that their child continued to experience severe restrictions in daily life, despite developmental challenges persisting (e.g., being underweight). Caregiver satisfaction with the intervention was high, with 93.3 % indicating being perfectly or well-satisfied with their child’s treatment.
Wilcocks et al., 2022 (United States of America) [44]	N = 30 (46.7 % female)	N = 11 caregivers (45.5 % female; M_age_=1.4, *SD*=0.7)	N = 19 caregivers of children with common gastrointestinal complaints (47.4 % female; M_age_=1.1, *SD*=0.9)	Colonic IPO (1) Gastroschisis (1) HD (1) NEC (5) Various Colonic and Intestinal Atresias (3)	SF−36	Overall QoL scores did not differ significantly between caregivers of children with SBS and their counterparts with healthy children (t(28) = −1.82, *p* = .079. However, emotional functioning was markedly lower for SBS caregivers compared to controls (M=60.73, *SD*=22.97 for SBS caregivers compared to M=80.00, SD=17.13 for reference group; *p* = .014). In qualitative interviews, caregivers endorsed heightened symptoms of stress and anxiety related to navigating the complexities of their child’s treatment as well as stress related to their parenting role, while noting the positive impact of having available social support from their child’s medical team, their family, and their peers.
Wong et al., 2022 (United States of America) [45]	N = 349	N = 66 (32 % female; Median_age_=8, IQR: 6–10).	N = 280 children with gastrointestinal conditions as well as healthy controls and SBS children	Gastroschisis (23) NEC (16) Other (14) Atresia (11) Midgut Volvulus (5) Original sample was N = 69 with three unretained participants.	PedsQL 4.0 Generic Core Scales	Self-reported health-related QoL was lower in children with IF compared to healthy children, with QoL scores higher for older children. Patients were noted to endorse QoL scores that were between 10 and 20 points lower than their healthy counterparts in terms of emotional, social, and school functioning. Further, children with IF reported significantly lower health-related QoL than even those children with chronic or gastrointestinal illnesses, across QoL dimensions, including physical, psychosocial, emotional, social, and school.
Zhang et al., 2023 (China) [46]	N = 443 for bowel function outcome analysis N = 138 for QoL analysis	N = 199 for bowel function N = 43 for QoL (26.6 % female; M_age_=84.4 months, *SD*=30.7)	N = 244 healthy controls N = 96 for QoL (91.6 months)	HD (138)	Rintala BFS PedsQL	IF children report significantly lower QoL than controls (*p* = .01), with considerably more frequent loss of educational activities (e.g., school absences; *p* = .005). Bowel functioning was significantly lower in the exposed sample compared to the control sample (M_score_=17.99, *SD*=2.479). Significant factors impacting bowel function included fecal soiling and accidents.

a Hirschsprung’s Disease

b Necrotizing enterocolitis

c Anorectal Malformations

d Chronic Intestinal Pseudo-obstruction

e Intestinal Pseudo-Obstruction

f Microvillus Inclusion Disease

g Short Bowel Syndrome

### Quality of life

#### Quality of life measures

Table 2 presents the unique standardized measures that were used to operationalize QoL in the included studies. The most common QoL measure used was the Pediatric Quality of Life Inventory; PedsQL [47]. Fourteen studies utilized the PedsQL Generic Core Scales [18], [24], [26], [28], [29], [30], [31], [32], [33], [34], [36], [38], [45], [46] and seven [15], [26], [30], [31], [34], [36], [42] used modules of the PedsQL such as the PedsQL: Family Impact [48], Infant [49], and Gastrointestinal Symptom Module [50]. Regarding disease-specific QoL, the Rintala Bowel Function Score System [51] was most often administered (*n* = 3 articles). Along with standardized QoL measures, some studies adapted existing measures to capture disease-specific QoL, such as Short-Bowel Syndrome [19], or unique circumstances, like the COVID-19 pandemic [25]. One study [39] conceptualized aspects of QoL through measures assessing specific psychological symptoms, such as the State-Trait Anxiety Inventory [52] and Psychological General Well-being Index [53].

**Table 2:** Standardized quality of life measures in pediatric intestinal failure studies.

**Measure**	**Description**	**Measure citation**	**Relevant Studies**
Baxter HPN-QoL questionnaire	This is a 48-item adult questionnaire, adapted for use in pediatric populations. It is comprised of functional scales (i.e., general health, ability to travel, physical function, coping, ability to eat/drink, emotional function), home parenteral nutrition items (i.e., nutrition team, ambulatory pump), symptom scales (i.e., body image, immobility, fatigue, sleep pattern, gastrointestinal symptoms, pain, presence of stoma, financial issues), and a QoL numerical rating scale.	J.P. Baxter, P.M. Fayers, A.W. McKinlay,. The clinical and psychometric validation of a questionnaire to assess the quality of life of adult patients treated with long-term parenteral nutrition. Journal of Parenteral and Enteral Nutrition 2009;34(2): 131–42. doi:10.1177/0148607109348612	[40]
Rintala Bowel Function Score (BFS) system	This is a 7-item measure capturing QoL information pertaining to bowel functioning (e.g., urge to defecate, ability to hold back defecation, constipation, social problems, etc.).	R.J. Rintala, H Lindahl. Is normal bowel function possible after repair of intermediate and high anorectal malformations? J Pediatr Surg 1995; 30(3): 491–4.doi:10.1016/0022–3468	[16], [18], [46]
Child Health Questionnaire (CHQ-CF87)	This is an 87-item survey assessing health-related QoL among children, accounting for global health, physical functioning, limitations in work, school, and activities with friends, mental health, behavioural difficulties, emotional impact, self-esteem, and family cohesion, among other variables.	H Raat, G.J. Bonsel, M.L Essink-Bot, J.M. Landgraf, R.J.B.J. Gemke. Reliability and validity of comprehensive health status measures in children. Journal of Clinical Epidemiology 2002; 55(1): 67–76.doi:10.1016/s0895–4356	[27], [35], [37]
DUX−25	This is a 25-item survey measuring the health-related QoL of children suffering from chronic diseases across four dimensions, including: physical, emotional, social, and home functioning. There are self-and-proxy report versions available.	M.M.P. Kolsteren, H.M. Koopman, G Schalekamp, M.L. Mearin. Health-related quality of life in children with celiac disease. The Journal of Pediatrics 2001; 138(4): 593–5. doi:10.1067/mpd.2001.111504	[24]
KLIK (kwaliteit van leven in kaart [Dutch Acronym for Quality of Life in Clinical Practice]	Dutch language questionnaire tracking health-related QoL and fatigue outcomes among patients on parenteral nutrition. An adult self-report version is also available.	Kwaliteit van Leven in Kaart (assessed 2025 May 6). Available from: https://www.hetklikt.nu/	[29]
Hirschsprung Disease/Anorectal Malformation Quality of Life Questionnaire (HAQL)	This is a 38-item questionnaire for children and adolescents, capturing health-related QoL across dimensions related to bowel functioning (e.g., fecal incontinence) as well as social, emotional, and physical functioning. There are self-and-proxy report versions available.	M.J. Hanneman, M.A. Sprangers, E.L. De Mik, E.L. van Heurn, Z.J. De Langen, N Looyaard, et al.. Quality of life in patients with anorectal malformation or Hirschsprung’s disease. Diseases of the Colon & Rectum 2001; 44(11):1650–60.doi:10.1007/bf02234386	[21], [22]
KIDSCREEN−52	This is a 52-item measure capturing health-related QoL across dimensions of physical and psychological well-being, moods, emotions, family functioning, social functioning, and school environment. It is based on the 10 Rasch-scale.	U Ravens-Sieberer, A Gosch, L Rajmil, M Erhart, J Bruil, M Power, et al.. The kidscreen−52 quality of life measure for children and adolescents: Psychometric results from a cross-cultural survey in 13 European countries. Value in Health 2008;11(4):645–58. doi:10.1111/j.1524–4733.2007.00291.x	[17]
Pediatric Quality of Life Inventory (PedsQL) - Generic Core Scales	This is a 23-item survey capturing physical, emotional, social, and school functioning. It is used with both healthy and chronically ill pediatric populations.	J.W. Varni, M Seid, T.S. Knight, K Uzark, I.S. Szer. The PedsQL 4.0 generic core scales: Sensitivity, responsiveness, and impact on clinical decision-making. J Behav Med 2002; 25(2):175–93.doi:10.1023/a:1014836921812	[18], [24], [26], [28], [29], [30], [31], [32], [33], [34], [36], [38], [45], [46]
Pediatric Quality of Life (PedsQL) - Family Impact Module (FIM)	This is a 36-item questionnaire assessing parent-and-self reported functioning across domains of physical, emotional, social, and cognitive functioning. As well, impact on family relationships, daily activities, communication, and level of worry are also captured.	J.W. Varni, S.A. Sherman, T.M. Burwinkle, P.E. Dickinson, P Dixon. The PedsQL family impact module: Preliminary reliability and validity. Health and Quality of Life Outcomes 2004; 2(1). doi:10.1186/1477–7525–2–55	[15], [31], [34], [36]
Pediatric Quality of Life Inventory (PedsQL) - Gastrointestinal Symptoms Scale	This is a 74-item measure capturing gastrointestinal symptoms across fourteen scales, including constipation, nausea, food and drink limits, stomach pain, and trouble swallowing. There are child self-report and caregiver proxy-report formats available dependent on child age (e.g., proxy report for children aged 2–4; parallel, specific versions for children and caregivers from ages 5–18 years).	J.W. Varni, C.B. Bendo, J Denham, R.J. Shulman, M.M. Self, D.A. Neigut, et al.. PedsQL gastrointestinal symptoms module: Feasibility, reliability, and validity. J Pediatr Gastroenterol Nutr 2014; 59(3):347–55. doi:10.1097/MPG.0000000000000414	[26], [42]
Pediatric Quality of Life Inventory (PedsQL) - Infant Scale	This is a 36 (1–12 months) or 45 (13–24 months) item survey measuring infant physical, emotional, social, and cognitive functioning.	J.W. Varni, C.A. Limbers, K Neighbors, K Schulz, J.E.C. Lieu, R.W. Heffer, et al.. The PedsQL™ infant scales: Feasibility, internal consistency reliability, and validity in healthy and ill infants. Qual Life Res Int J Qual Life Asp Treat Care Rehabil 2011; 20(1) :45–55. doi:10.1007/s11136–010–9730–5	[30], [36]
Quality of Life Scoring Criteria for children with fecal incontinence	This is a 6-item survey capturing challenges related to fecal incontinence in pediatric populations, such as incontinence, school absence, food restriction, peer rejection. There are child self-report and caregiver proxy-report versions available.	Y Bai, H Chen, J Hao, Y Huang, W Wang. Long-term outcome and quality of life after the Swenson procedure for Hirschsprung’s disease. J Pediatr Surg 2002; 37(4):639–42.doi:10.1053/jpsu.2002.31625	[14]
TNO-AZL Questionnaires for Children’s Health-Related Quality of Life (TACQOL)	This is a 56 (8–11 years) or 86 (12–16 years) item survey assessing seven subscales such as physical, motor, cognitive, social, emotional, and adaptive functioning. There are self-and-proxy report versions available.	T Vogels, G.H. Verrips, S.P. Verloove-Vanhorick, M Fekkes, R.P. Kamphuis, H.M. Koopman, et al.. Measuring health-related quality of life in children: The development of the TACQOL parent form. Quality of Life Research 1998; 7(5) :57–65. doi:10.1023/a:1008848218806	[21], [22], [23], [41]

#### Pediatric intestinal failure and quality of life

**Fig. 2:**
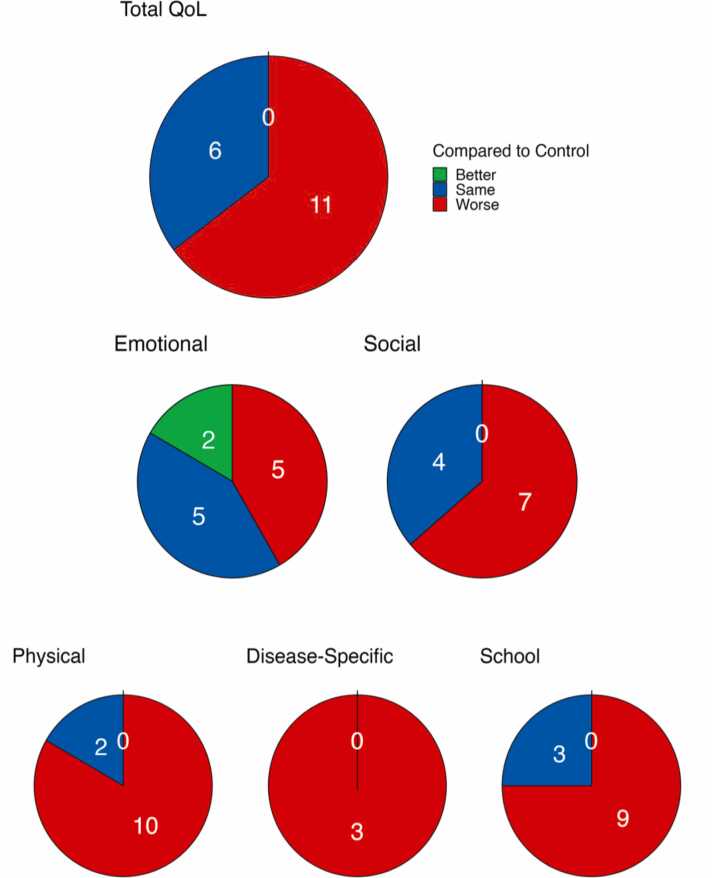
Self-reported quality of life among pediatric IF patients compared to controls.

All 33 studies evaluated associations between pediatric IF and QoL: *n* = 32 through quantitative methods and *n* = 1 [20] through qualitative focus interviews. Fig. 2 shows the direction of study findings compared to controls.

### Patient self-reported QoL

Ten findings across 33 studies indicated that self-reported health-related QoL in the IF sample was significantly lower than healthy control participants [14], [18], [26], [27], [29], [30], [33], [34], [38], [45]; with one noting worse self-reported QoL relative to a health-risk population (gastrointestinal patients) (GI)) [45]. Child-reported QoL was not significantly different than controls in six instances [17], [22], [26], [28], [32], [45], and better than controls in none.

### Physical functioning

With two exceptions [17], [32], nine studies indicated worse self-reported physical functioning relative to healthy controls [26], [27], [28], [29], [30], [33], [34], [38], [45] and one relative to a pediatric intestinal transplant population [35].

### Disease-specific functioning

Three studies [17], [18], [32] noted worse bowel functioning among IF children with Hirschsprung’s Disease, compared to controls. Notably, poorer bowel functioning was associated with fecal incontinence and constipation, both of which had deleterious impacts on social and school functioning.

### Social functioning

Four studies noted no significant differences between self-reported social functioning among IF children and controls [17], [26], [28], [33]. Conversely, seven findings reported that IF children perceived their social functioning to be worse than their control counterparts: six comparing to healthy controls [29], [30], [34], [38], [45], and one to health risk GI populations [45].

### School functioning

With three exceptions, which reported no significant difference between self-reported school functioning and controls [17], [28], [32], nine findings (six compared to healthy controls, and three compared to chronically-ill samples) noted self-reported school functioning as worse than controls [26], [28], [29], [33], [34], [38], [45]. School absence as well as negative school attitudes reportedly undergirded poorer school functioning among IF patients.

### Emotional functioning

Findings were mixed in terms of self-reported emotional functioning (compared to controls); five studies noted worse emotional functioning for the IF sample [26], [30], [38], [42], [45], five identified similar scores [17], [28], [29], [33], [34] and two reported better emotional functioning in the IF sample, (one of which drew comparisons to a post-intestinal transplant group) [35]. The ability to adapt and cope with illness was qualitatively identified as promoting resilience in the IF groups [20].

Overall, pediatric IF patients reported worse outcomes across all domains of QoL, apart from emotional functioning. Table 3 documents QoL domains consistently captured within pediatric IF research.

**Table 3:** QoL domains in pediatric intestinal failure.

**Domains**	**Subdomains**	**Examples**
**Overall QoL**	Total QoL	Proxy / self-report measures of total QoL
**Physical**	Disease symptoms and severity Medical comorbidity	IF etiology, constipation, fecal incontinence, stoma
**Emotional**	Mental health Self-image	Anxiety, self-esteem, stress, body image
**Social**	Family functioning and caregiver burden Peer relationships	Caregiver QoL and financial challenges Restricted social opportunities, social judgement linked to illness
**School**	Educational Cognitive deficits	School absences, negative school attitude Learning and attentional difficulties

### Caregiver QoL

Three studies [15], [41], [44] considered caregiver health-related QoL, with mixed findings. Belza and colleagues [15] found that caregivers of children with IF on PN reported worse total QoL relative to caregivers of healthy children, with significantly worse outcomes across QoL domains. Caregivers also reported increased stress, anxiety, and depression, with stress highly correlated to worse health-related QoL; greater caregiver anxiety and stress was also documented in one other study [39]. In contrast, van Oers et al. [41] evaluated QoL among caregivers of children with IF on home PN and identified no significant differences between caregivers’ health-related QoL and that of control caregivers. However, caregivers in this study generally reported having less meaningful social connections compared to controls. Findings from Wilcocks and colleagues [44] aligned with these results, noting no significant differences in QoL among caregivers of IF children and healthy control caregivers. However, emotional functioning was considerably lower for IF sample caregivers, and notably, all other QoL domains trended towards being worse relative to caregivers of healthy children. Managing the demands of home PN treatment was a notable source of caregiver stress [39], [41].

Two studies [31], [34] evaluated family functioning through the PedsQL: Family Impact Model (FIM) questionnaire. Across both studies, caregivers of children with IF reported worse outcomes among family functioning, and worry. However, IF families reportedly had better scores within the family relationships domain of the FIM compared to a reference sample of healthy and chronically ill families [31].

### Cross-informant effects

**Fig. 3:**
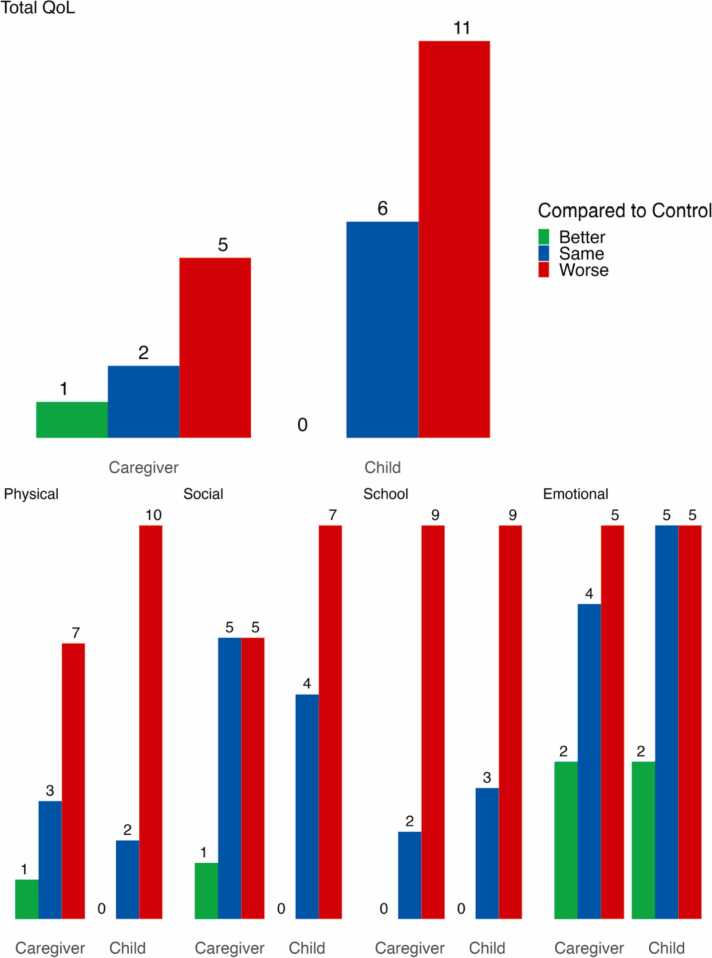
Parent-proxy and child-self reported QoL outcomes compared to controls.

Fig. 3 displays the frequency of findings for caregiver proxy and child self-reported QoL outcomes, compared to controls. Across studies, caregivers, like children, reported lower health-related QoL compared to controls [26], [28], [29], [33], [46]. However, in one study [38], caregivers reported better QoL for their children with IF compared to healthy controls, with two studies [24], [32] identifying no differences between IF QoL and control outcomes. This trend was also observed across the physical, social, and emotional domains of QoL.

Alongside general findings, four studies explicitly compared proxy caregiver and child outcomes on QoL domains. Based on averages, Silva and colleagues [38] reported that caregivers indicated better QoL scores than children across all domains except social and physical functioning. Corroborating these findings, caregivers in Hartman and colleagues [23] study indicated that their children with IF experienced more positive emotions than the children indicated themselves. Caregivers also rated physical functioning more highly for both their healthy child and their child with IF. Further, Neuvonen and colleagues [32] found caregivers reported that their children experienced fewer negative emotions than the children did themselves. At the same time, across three studies [23], children with IF reported better overall health-related QoL than their caregivers. Interestingly, caregivers of children with IF agree more with their children about their health-related QoL than caregivers of healthy children do with their offspring [23].

### Age and quality of life

Seven studies examined age-related differences in QoL, comparing children (typically 8–12 years) and adolescents (13–18 years) [21], [22], [23], [29], [30], [36], [45]. Adolescents reported higher QoL and perceived self-competence than healthy controls but had poorer QoL compared to younger IF children in the sample [22]. Relative to healthy controls, teenagers did not report different scores across QoL domains, save for lower physical functioning [29], [30]. In contrast, children reported lower QoL scores in every domain except emotional functioning [29]. Relatedly, teens indicated significantly poorer self-esteem and school attitudes compared to children [20], [21]. Interestingly, comparing proxy report of infants and toddler (1 month to 4.99 years) to older children (5−12 years), Neam et al., [30] found that the infants and toddlers had the highest health-related QoL, with QoL declining in the 5–12 age range, especially within the domain of school functioning. Children (aged 5–12 years) reported significantly worse QoL across all domains except social functioning, as compared to healthy controls.

### Risk and protective factors in QoL

Of the included studies, *n* = 24 identified physical, emotional, social, cognitive, and disease-specific risk factors associated with lower QoL among IF patients. Conversely, only *n* = 10 studies [20], [21], [22], [23], [28], [32], [40] reported on protective factors for QoL in pediatric IF patients.

### Risk factors

Across studies, poorer bowel functioning frequently emerged as a risk factor for worse reported QoL, including fecal incontinence and constipation [14], [18], [27], [32], [39], [42]. Further, greater disease severity, complexity, frequent abdominal pain, and having longer remnant small bowel length were associated with lower QoL among children with IF [21], [22], [28], [45]. Other medical risk factors included PN dependence (especially for communication and school attendance), major disease comorbidities, greater number of out-patient hospital visits, and higher recent (within the past year) in-patient hospital admissions [15], [21], [22]. Early developmental factors such as birth prematurity, neonatal IF onset, and developmental delay were related to poorer QoL outcomes [24], [29], [30]. QoL was also lower in IF patients reporting peer victimization, negative social comparison, negative school attitudes, and restrictions to their social life [20]. Family factors such as living in a single-parent home, greater parental stress and anxiety, and higher parent perceived vulnerability were associated with lower patient QoL [28], [41]. In addition, having cognitive difficulties, negative self-image, and lower perceived athletic competency, as well as sociodemographic factors like identifying as a girl and being from a mixed-race background, were identified as risk factors for lower QoL [21], [22], [35], [36].

### Protective factors

Positive, accepting social relationships with adults and peers were linked to higher QoL among IF patients [20]. Relatedly, support from siblings and the presence of pets in a child’s life were also linked to greater QoL [20]. Individual protective factors included having a positive self-image, an adequate understanding of their disease and treatment, participation in childhood activities, higher perceived athletic competency, greater self-esteem, positive school attitudes, and more frequent school attendance [20], [21], [22], [23]. As previously mentioned, older children typically reported better QoL compared to controls. Medical factors, such as the absence of a stoma, were associated with greater patient ability to engage in social and leisure activities, linked to higher QoL [40]. Also, longer time since PN weaning, greater time intervals since the last bowel operation, and fewer hospitalizations were linked to lower parental stress [28]. Protective family factors also included living in a two-parent household [35].

## Discussion

### Summary of evidence

This scoping review evaluated QoL among pediatric IF samples, with a novel focus on organizing the QoL measures that have been employed within the pediatric IF literature. Research specifically addressing QoL in this population is limited. This study, which spanned the past six decades, identified only 33 articles that explicitly defined, operationalized, and measured QoL in pediatric IF patients. Demonstrating this as a burgeoning field, 73 % of these studies were published within the past decade.

The most common tool used to measure QoL was through standardized, self-and-caregiver-reported questionnaires. The PedsQL emerged as the instrument used most often [18], [24], [26], [28], [29], [30], [31], [32], [33], [34], [36], [38], [45], [46]. Studies typically captured global QoL, including physical, emotional, social, and school functioning, and in some instances included additional domains such as communication, family, and cognitive functioning [15], [23], [34]. Importantly, the PedsQL has been identified by caregivers, and within the broader literature, as not specific or sensitive enough to capture the nuanced needs of children with IF [36], [54], emphasizing the need to develop a disease-specific tool. Further, most QoL tools focus on capturing individual functioning, but do not measure other vital aspects that inform QoL, such as stress management and coping strategies. Given the salience of the link between the use of coping strategies with greater QoL [55], [56], open-ended items that allow children and caregivers to note how they feel about different QoL domains may be of use in an IF-specific QoL tool.

Across studies, a common consensus emerged: IF patients self-report worse health-related QoL across domains. Further, caregivers of patients with IF also note higher rates of stress, depression, and anxiety symptoms, which negatively impact their own QoL [15], [44]. We can speculate that IF is a condition that has a deleterious whole-family effect, with lower QoL experienced by both the child and their family. Poorer QoL among this population is associated with worse mental health outcomes, such as increased anxiety [42]. School-aged populations report significantly more emotional problems than controls; parents and teachers report similarly clinically elevated emotional problems among this age-group [42]. Over time, mental health challenges can have a negative impact on essential facets of health, such as medication adherence, an aspect of health management that children and adolescents often struggle with especially as they transition to independent living and adult medical programs [57], [58]. Youth with IF may additionally be at greater risk of trauma-related symptoms [42] and adopting non-adaptive coping strategies, which has been observed in pediatric solid organ transplant populations, and negatively impacts treatment adherence [59], [60]. Further, among this population, self-esteem issues in relation to their illness and physical appearance contributes to worse mental health outcomes and poorer QoL [61]. The increasing prevalence of mental health challenges within youth IF patients, highlight the need to integrate mental health specialists within multidisciplinary intestinal failure teams. Specialists, like health psychologists and medical social workers, who are aware of the medical complexities unique to IF patients, should be embedded into interdisciplinary teams, as community-based professionals may not fully appreciate IF-linked medical complexities and subsequent psychological vulnerabilities.

School functioning is consistently identified by children and caregivers as a source of low QoL, impacted by school absence (often due to medical needs) or negative school attitudes. The higher prevalence of learning and attentional difficulties [62], [63] in this population may negatively impact academic success. Therefore, tracking patient cognitive functioning over time, and providing relevant diagnoses and recommendations for tailored educational supports will be important. A neuro/psychologist embedded within the IF team could support this type of assessment. Academic underachievement may also make youth with IF vulnerable to social difficulties [64], which alongside ‘visible’ differences (such as being on PN, being shorter than peers, or having scars), may further lower self-esteem within this population [65].

Consistent with other populations [66], proxy-report diverged from self-report when providing QoL evaluations, with caregivers indicating that children have worse QoL than children did themselves [28], [45]. Previous evidence has observed that when caregivers report low health-related QoL, often in relation to high caregiver burden, they also evaluate their child’s QoL as worse than the child themselves [67]. Caregiver burden is exacerbated for IF families [68], which may negatively impact QoL appraisal. Given this, there is a need to assess QoL using multiple informants, to ensure a holistic picture of QoL. Clinical teams should consider incorporating caregiver report in a strategic manner, particularly as a vehicle to facilitate familial supports (e.g., target caregiver mental health through referrals to local services or peer support networks).

Our findings also demonstrated age-related QoL differences. Adolescents with IF generally reported worse QoL compared to children with IF, despite noting similar QoL outcomes to healthy teenagers [21], [22]. Adolescents with IF also reported worse self-esteem and school attitudes compared to IF children [21]. The worsening of adolescent QoL from childhood onwards, although typical, is cause for concern. The evidence emphasizes the importance of providing psychological support for youth with IF at all ages, but specifically targeting the teen years, to develop age-appropriate coping skills to manage disease and daily life stressors.

Lastly, our review identifies several medical, social, and sociodemographic factors that may be useful clinical indicators for proactive intervention. At-risk families (e.g., single-parent household with a complex disease) could be provided with caregiver psychoeducation [69], financial support, mental health screening, and opportunities to engage with peers (e.g., through hospital sponsored camp experiences, as well as peer and caregiver support groups).

### Limitations

Limitations of this study include a search that was restricted to English-language articles, narrowing our ability to comment on untranslated, regional adaptations of QoL measures. Additionally, as we aimed to investigate how QoL is measured in the literature, we required that articles explicitly stated their constructs as representing QoL. There may be studies omitted that implicitly capture elements of QoL. This variability highlights the need for consensus regarding how QoL measurement is defined and implemented.

## Conclusions

This review is a first step in highlighting approaches to measuring QoL in IF patients and their families. Diverse and significant QoL needs among children with IF and their families were identified. Findings can inform a cohesive and consistent measurement of QoL among IF patients, as well as inform precise, targeted intervention for maximal life fulfillment in pediatric IF patients and their families.

## Patient's / guardian's consent

Not applicable.

## Ethical clearance

Not required.

## Funding

This research did not receive any specific grant from funding agencies in the public, commercial or not-for-profit sectors.

## CRediT authorship contribution statement

**Fatima Wasif:** Methodology, Validation, Data curation, Software, Visualization, Writing – review & editing, Writing – original draft. **Anna Gold:** Conceptualization, Methodology, Validation, Writing – review & editing, Data curation, Supervision, Writing – original draft. **Dylan Johnson:** Investigation, Validation, Writing – review & editing, Conceptualization, Methodology, Writing – original draft.

## Declaration of Competing Interest

None.

## Footnotes

Appendix A Supplementary data associated with this article can be found in the online version at doi:10.1016/j.intf.2025.100071.
